# SLC24A-mediated calcium exchange as an indispensable component of the diatom cell density-driven signaling pathway

**DOI:** 10.1093/ismejo/wrae039

**Published:** 2024-03-08

**Authors:** Xuehua Liu, Zhicheng Zuo, Xiujun Xie, Shan Gao, Songcui Wu, Wenhui Gu, Guangce Wang

**Affiliations:** CAS and Shandong Province Key Laboratory of Experimental Marine Biology, Institute of Oceanology, Chinese Academy of Sciences, Qingdao 266404, Shandong Province, China; Key Laboratory of Breeding Biotechnology and Sustainable Aquaculture, Chinese Academy of Sciences, Qingdao 266404, Shandong Province, China; Laboratory for Marine Biology and Biotechnology, Qingdao Marine Science and Technology Center, Qingdao 266237, Shandong Province, China; College of Chemistry and Chemical Engineering, Shanghai University of Engineering Science, Shanghai 201620, China; Shanghai Frontiers Science Research Center for Druggability of Cardiovascular Noncoding RNA, Institute for Frontier Medical Technology, Shanghai University of Engineering Science, Shanghai 201620, China; CAS and Shandong Province Key Laboratory of Experimental Marine Biology, Institute of Oceanology, Chinese Academy of Sciences, Qingdao 266404, Shandong Province, China; Key Laboratory of Breeding Biotechnology and Sustainable Aquaculture, Chinese Academy of Sciences, Qingdao 266404, Shandong Province, China; Laboratory for Marine Biology and Biotechnology, Qingdao Marine Science and Technology Center, Qingdao 266237, Shandong Province, China; CAS and Shandong Province Key Laboratory of Experimental Marine Biology, Institute of Oceanology, Chinese Academy of Sciences, Qingdao 266404, Shandong Province, China; Key Laboratory of Breeding Biotechnology and Sustainable Aquaculture, Chinese Academy of Sciences, Qingdao 266404, Shandong Province, China; Laboratory for Marine Biology and Biotechnology, Qingdao Marine Science and Technology Center, Qingdao 266237, Shandong Province, China; CAS and Shandong Province Key Laboratory of Experimental Marine Biology, Institute of Oceanology, Chinese Academy of Sciences, Qingdao 266404, Shandong Province, China; Key Laboratory of Breeding Biotechnology and Sustainable Aquaculture, Chinese Academy of Sciences, Qingdao 266404, Shandong Province, China; Laboratory for Marine Biology and Biotechnology, Qingdao Marine Science and Technology Center, Qingdao 266237, Shandong Province, China; CAS and Shandong Province Key Laboratory of Experimental Marine Biology, Institute of Oceanology, Chinese Academy of Sciences, Qingdao 266404, Shandong Province, China; Key Laboratory of Breeding Biotechnology and Sustainable Aquaculture, Chinese Academy of Sciences, Qingdao 266404, Shandong Province, China; Laboratory for Marine Biology and Biotechnology, Qingdao Marine Science and Technology Center, Qingdao 266237, Shandong Province, China; CAS and Shandong Province Key Laboratory of Experimental Marine Biology, Institute of Oceanology, Chinese Academy of Sciences, Qingdao 266404, Shandong Province, China; Key Laboratory of Breeding Biotechnology and Sustainable Aquaculture, Chinese Academy of Sciences, Qingdao 266404, Shandong Province, China; Laboratory for Marine Biology and Biotechnology, Qingdao Marine Science and Technology Center, Qingdao 266237, Shandong Province, China

**Keywords:** diatom cell density, density signals, Ca^2+^, SLC24A, Phaeodactylum tricornutum

## Abstract

Diatom bloom is characterized by a rapid increase of population density. Perception of population density and physiological responses can significantly influence their survival strategies, subsequently impacting bloom fate. The population density itself can serve as a signal, which is perceived through chemical signals or chlorophyll fluorescence signals triggered by high cell density, and their intracellular signaling mechanisms remain to be elucidated. In this study, we focused on the model diatom, *Phaeodactylum tricornutum*, and designed an orthogonal experiment involving varying cell densities and light conditions, to stimulate the release of chemical signals and light-induced chlorophyll fluorescence signals. Utilizing RNA-Seq and Weighted Gene Co-expression Network Analysis, we identified four gene clusters displaying density-dependent expression patterns. Within these, a potential hub gene, *PtSLC24A*, encoding a Na^+^/Ca^2+^ exchanger, was identified. Based on molecular genetics, cellular physiology, computational structural biology, and *in situ* oceanic data, we propose a potential intracellular signaling mechanism related to cell density in marine diatoms using Ca^2+^: upon sensing population density signals mediated by chemical cues, the membrane-bound *PtSLC24A* facilitates the efflux of Ca^2+^ to maintain specific intracellular calcium levels, allowing the transduction of intracellular density signals, subsequently regulating physiological responses, including cell apoptosis, ultimately affecting algal blooms fate. These findings shed light on the calcium-mediated intracellular signaling mechanism of marine diatoms to changing population densities, and enhances our understanding of diatom bloom dynamics and their ecological implications.

## Introduction

Diatoms constitute at least 20% of the global productivity, by providing 40% of marine primary productivity, exerting a pivotal influence on biogeochemical cycles [1, 2]. The abundant diatom biomass predominantly stems from the occurrence of explosive diatom blooms. The scale and extension of blooms are collectively conditioned by diverse factors, deriving from surrounding environment and cell population. Surrounding environment factors, such as nutrients, trace elements, temperature, viruses, predators, have been demonstrated for their important role in affecting cell physiology, thereby affecting the magnitude and dynamics of algal blooms [3–8]. In addition to these factors, cell density fluctuation during blooms could serve as a direct signaling factor affecting the physiological processes of diatoms.

During the initiation and expansion of diatom blooms, the cell density could reach more than 10^6^ cells L^−1^ [9–11], sometimes as high as 3 × 10^8^ cells L^−1^ [12]. Density-dependent physiological responses have been widely appreciated in diatom [13–17], dinoflagellates, cyanobacteria, and haptophyte for their regulatory role in resting spore formation, grazing responses, sexual reproduction, nutrient competition, etc. [18–23]. In certain instances, density-dependent but nutrient-independent interactions could trigger programmed cell death (PCD) between the patch-forming dinoflagellate, *Peridinium gatunense*, and the toxic cyanobacterium, *Microcystis* sp*.* [22], which has been proposed to influence substantial phytoplankton turnover and blooms termination [24, 25]. It is evident that population density signals significantly impact critical physiological processes within cells, thereby playing a vital role in shaping bloom fate. Further investigation into the density-related intracellular signaling mechanisms in algal blooms holds promise for yielding fresh insights into the bloom dynamics.

As a well-demonstrated example of density signaling mechanism, quorum sensing in bacteria is able to assess and respond to population density changes through the production, release, and detection of extracellular signaling information known as autoinducers [26]. The recognition of autoinducers subsequently initiates the activation of intracellular second messenger signaling systems (Ca^2+^, cAMP, etc.), consequently regulating gene expression [27–29]. Similarly, in diatoms, extracellular signaling information regarding population density could be transmitted and sensed by diatom cells, in the form of chemical cues [15–17]. Diatom could regulate spore formation [15], oxylipin production [16], and sexual reproduction [17] in a density-dependent manner, but the types of these chemical signals are unclear. In the intracellular signaling process, some biotic-derived infochemicals, such as 2E, 4E/Z-decadienal (DD), and other bacterial quorum-sensing molecules, could induce the intracellular Ca^2+^ signaling and NO responses [30–32]. Those fluctuated intracellular Ca^2+^ signaling-induced extracellular bioelectricity is supposed to be a kind of paracrine signaling in microalgae communication [23]. Compared with the accumulated evidence regarding density-sensing mechanisms in bacteria, research in diatoms lags significantly behind because of the complexity of oceanic environment. Especially, the intracellular signaling generated from the density fluctuation within diatom population remains elusive, and the involvement of Ca^2+^ in secondary signaling process requires verification experimentally.

In contrast to quorum sensing in bacteria which relies only on chemical signals, diatom cells possess the unique ability to employ spontaneous chlorophyll fluorescence as a light signal to perceive changes in population density [33, 34]. In ocean environment, chlorophyll fluorescence derived from phytoplankton contributes significantly to red and far-red irradiation underwater [35, 36]. Our recent study demonstrated that *Phaeodactylum tricornutum* could perceive enhanced chlorophyll fluorescence derived from neighboring cells under high cell density to facilitate iron assimilation [33]. Font-Muñoz also reported this signal and pointed out that sunlight-induced fluorescence signals were used in pelagic diatoms communication to coordinate their population behavior [34]. The chlorophyll fluorescence signal, serving as a red-light signal, is possibly mediated by the red-light receptor, phytochrome. Though characterized in the marine diatoms *P. tricornutum* and *Thalassiosira pseudonana*, phytochrome does not directly involved in red light signaling in *P. tricornutum* [36]. Besides, conserved components of the photochrome signaling pathway, such as COP1, HY5, etc. have not yet been identified in the *P. tricornutum* genome. Thus, there is a considerable challenge in investigating the chlorophyll fluorescence signal from the perspective of phytochrome. Moreover, recent investigations have unveiled the involvement of intracellular Ca^2+^ signaling in phytochrome-dependent regulation downstream of phytochrome [37–39]. This revelation suggests a promising avenue for initiating research, focusing on intracellular Ca^2+^ signaling, to elucidate the intracellular signaling of density-dependent chlorophyll fluorescence signal.

In this study, we aimed to investigate the key player in the intracellular signaling of diatom, after sensing the density information carried by chemical cues or chlorophyll fluorescence signal. Because the chlorophyll fluorescence was excited by sunlight, with fluorescence intensity being directly proportional to cell density, genes regulated by chlorophyll fluorescence display density-dependent expression pattern under light, whereas remaining unaltered in dark [33]. Conversely, the density-dependent expression of genes under the influence of chemical signals was independent of light. Thus, we designed an orthogonal experiment involving varying cell densities and light conditions to stimulate the release of chemical signals and chlorophyll fluorescence. Then, comparative transcriptome and weighted gene co-expression network analysis (WGCNA) were performed to identify potential genes involved in the density-dependent intracellular signal transduction. The potential regulatory role of identified Na^+^/Ca^2+^ exchange domain containing genes was explored using CRISPR/Cas9 editing and queried in the Tara Oceans eukaryotes gene atlas. Based on molecular genetics, cellular physiology, computational structural biology, and *in situ* oceanic data, this study attempts to investigate the molecular mechanisms in density-related intracellular signaling.

## Materials and methods

### Cell culture and treatment conditions

*P. tricornutum* were obtained from the Institute of Hydrobiology, Chinese Academy of Sciences and grown in sterile artificial seawater containing f/2 medium at 20°C ± 1°C. The cultures were illuminated by fluorescent white lamps at an intensity of 80 μE/m^2^/s under a light/dark cycle of 12/12 h.

During the diatom blooms in oceans, the cell density could reach as high as 3 × 10^5^ cells/ml [12]. However, it is very difficult to obtain enough biomass for further experiment if the culture biomass completely simulates the density changes in natural diatom blooms, because of many practical difficulties such as the requirement of large-scale cultivation, lighting devices, and cell harvesting. As mentioned in the Introduction section, the cell density fluctuation during diatoms blooms in ocean is sufficient to trigger a series of physiological responses [14, 16, 18, 19, 22]. Therefore, in this study, the cell density treatment was set at low (L), medium (M), and high (H) levels (1.5 × 10^6^ cells/ml, 3 × 10^6^ cells/ml, and 6 × 10^6^ cells/ml separately to simulate the cell density of the early, middle, and late stages of the cell growth curve), which was conducive to enrich the effect of cell density more significantly and find different expression genes and pathways.

**Figure 1:**
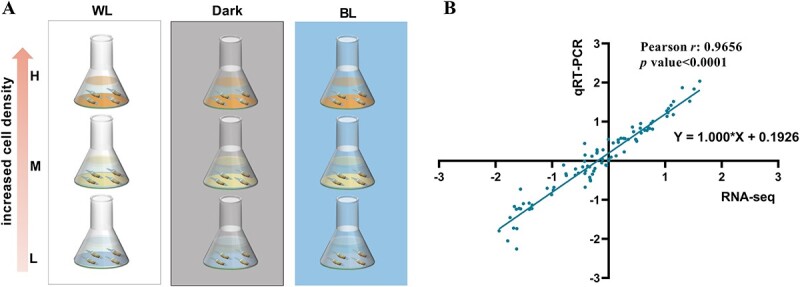
Overview of transcriptome analysis; (A) schematic illustration of experimental design; the cells at different cell density levels [low (L), medium (M), and high (H)] were illuminated under WL, BL, or dark conditions (Dark); (B) qRT-PCR validation of RNA-seq; a total of 11 genes were selected randomly to conduct the qRT-PCR verification; the expression level of every gene was normalized by the WL-L group; the X-axis is the value of log_10_(normalized relative expression derived from RNA-seq); the Y-axis is the value of log_10_(normalized relative expression derived from qRT-PCR); Pearson correlation analysis and linear regression were performed on the expression of 11 genes under 9 treatments, total 99 points.

The treatment conditions of cell density and light are depicted in Fig. 1A. For different cell density treatments, the algal cells were cultured under white fluorescent lamps (80 μE/m^2^/s) till the mid-exponential growth phase, and harvested by centrifugation at 2000 × *g* for 5 min. Then, the cells were inoculated into fresh medium and the cell densities were adjusted to low (1.5 × 10^6^ cells/ml), medium (3 × 10^6^ cells/ml), and high (6 × 10^6^ cells/ml). For different light treatments, the algal cells at various cell densities were cultured under blue light (BL, 460 nm, 80 μE/m^2^/s), white light (WL, 80 μE/m^2^/s), or dark for 24 h. After that, the cells were harvested by centrifugation at 6000 × *g* for 1 min and quickly frozen with liquid nitrogen.

### RNA extraction, sequencing, and annotation

Total RNA was extracted using Trizol reagent kit (Invitrogen, Carlsbad, CA) according to the manufacturer’s instructions. The mRNA was accessed by electrophoresis on 1% agarose gel and quantified by a NanoDrop 2000 spectrophotometer (Thermo Fisher Scientific Inc., Waltham, MA). Oligo(dT) beads (Epicentre) were used to enrich the mRNA. Then the enriched mRNA was fragmented into short fragments using fragmentation buffer and reverse transcripted into cDNA with random primers. Second-strand cDNA was synthesized by DNA polymerase I, RNase H, dNTP, and buffer. Then the cDNA fragments were purified with QiaQuick PCR extraction kit (Qiagen, Venlo, The Netherlands), end repaired, poly(A) added, and ligated to Illumina sequencing adapters. The ligation products were size selected by agarose gel electrophoresis, PCR amplified, and sequenced using Illumina Novaseq6000 by Gene Denovo Biotechnology Co. (Guangzhou, China).

To eliminate the effect of adapters and low-quality bases on the following assembly and analysis, reads obtained from sequencing machines were filtered by fastp (version 0.18.0) [40]. Reads that were filtered out included those containing adapters, or containing more than 10% of unknown nucleotides, or low-quality reads containing more than 50% of low quality (Q-value ≤20) bases. To remove residual rRNA reads, the high-quality reads were mapped to an rRNA sequence using alignment tool Bowtie2 (version 2.2.8) [41]. The paired-end clean reads were mapped to the reference genome (*P. tricornutum* CCAP 1055/1, assembly ASM15095v2) using HISAT2. 2.4 [42] with “-rna-strandness RF” and other parameters set as a default. Gene abundances were calculated and normalized to FPKM reads.

### Weighted gene co-expression network analysis

To construct the co-expression networks underlying different cell densities and light, WGCNA analysis was performed using an R package [43]. Transcripts with reads per kb per million (FPKM) count ≥1 were imported into WGCNA to construct co-expression modules using the automatic network construction function block wise Modules with default settings, except that the power was 8, TOM type was unsigned, weighted correlation threshold ≥0.85, and min module size was 50. Genes were clustered into 16 correlated modules. To find out biologically significant modules, module eigengenes were used to calculate the correlation coefficient with samples or sample traits. Intramodular connectivity and module correlation degree of each gene were calculated by R package of WGCNA [43], and genes with high connectivity tended to be hub genes which might have important functions. Correlation analysis was performed using module eigengene with data for specific traits or phenotypes. Pearson correlation between each gene and trait data under the module was also calculated for the most relevant module (positive correlation and negative correlation) corresponding to each phenotype data, and gene significance (GS) value was obtained.

For genes unique expressed in each module, Kyoto Encyclopedia of Genes and Genomes (KEGG) pathway enrichment analyses were conducted to analyze the biological functions of modules.

### Gene co-expression network visualization and hub genes identification

In the edge file generated by WGCNA, genes with the top 99 weight values were visualized as gene co-expression network by Cytoscape (v3.9.1) [44]. The cytoHubba plug-in Cytoscape was employed to identify potential hub genes in each module based on Maximal Clique Centrality (MCC) method. For the top six genes ranked by cytoHubba, we proceeded to comprehensively consider the module membership (MM) values and GS values associated with density traits. Within each module, we identified and retained three candidate hub genes that met the criteria of MM > 0.8 and GS > 0.2, prioritizing those with relatively higher MM values. These selected candidate hub genes were highlighted in the co-expression network.

### Quantitative real-time PCR

To verify the reliability and authenticity of RNA-seq, 11 unigenes were selected to detect their expression levels in different treatments using quantitative real-time polymerase chain reaction (qRT-PCR). About 1 μg RNA was used for reverse transcription using PrimeScript RT Reagent Kit (TaKaRa, Kyoto, Japan). The cDNA obtained was quantified by qRT-PCR using FastStart Essential DNA Green Master kit (Roche Diagnostics GmbH, Mannheim, Germany) and QuantStudio 1 System (Applied Biosystems, Foster City, CA). Relative gene expression levels were calculated following the comparative Ct method with the analysis formula 2^−△△Ct^. The internal control was the 30S ribosomal protein subunit gene (*RPS*) [45], and the primer information is listed in Supplementary Table S1. Pearson correlation analysis and linear regression were performed using GraphPad Prism 8.0.2 (www.graphpad.com).

To evaluate the gene expression level in *PtSLC24A* knockout lines, six unigenes were selected, and the qRT-PCR was performed as described above. The internal control was the *RPS*, and the primer information is listed in Supplementary Table S2.

### Computational modeling and simulations

The PtSLC24A protein falls within the Na^+^/Ca^2+^ exchanger (NCX) family, with a full length of 651 amino acid residues (UniPort entry: B7S441). It comprises a transmembrane (TM) domain and a large intracellular regulatory domain [46]. The TM domain is the functional unit for ion transport [46], and here its 3D model (covering residues 12–183 and 489–651) was created by AlphaFold Monomer v2.0 pipeline [47]. The TM domain was embedded in a POPC (1-Palmitoyl-2-oleoylphosphatidylcholine) bilayer membrane with explicit solvent of 150 mM NaCl. The Ca^2+^ was positioned at the S_Ca_ site with reference to the Ca^2+^-loaded crystal structure of NCX from *Methanococcus jannaschii* [48]. CHARMM-GUI [49] was employed to set up the protein-membrane system and to generate the inputs for molecular dynamics simulations. The built simulation box has a size of 95.2 × 95.2 × 102.8 Å^3^, containing 224 POPC lipids and 17 094 water molecules. The protein and lipids were described with the AMBER ff19SB and lipids21 force fields, respectively, with the TIP3P model for water [50, 51]. The Ca^2+^ was treated with the multisite ion mode developed by Saxena and Sept [52], whose accuracy has been exemplified in our previous studies [53–56]. The GPU-accelerated version of the AMBER22 *pmemd* engine [57] was harnessed to perform the simulations. The temperature was maintained at 298.15 K using Langevin thermostat, and the pressure was kept at 1.0 bar with Berendsen barostat. The simulation system reached stable after going through a sequence of energy minimization, heating, and equilibration stages, with diminishing distance/dihedral restraints on the solutes and the Ca^2+^ ion. Two independent simulations were then carried out, each extending up to 700 ns. The last 200 ns of the simulation trajectories were extracted for analysis.

### Construction of PtSLC24A knockout lines by CRISPR/Cas9

To construct the *PtSLC24A* gene knockout mutants, conjugative system for Cas9 editing was performed according to the protocol reported by Slattery *et al*. [58] with small modifications. Sequence of gRNAs targeting *PtSLC24A* in *P. tricornutum* were designed using CRISPOR.org web tool (http://crispor.tefor.net/) [59] (gRNA: TCTCTCGTTCTGGGGTCAGGCGG). After phosphorylating and annealing the ordered oligos, the gRNAs were cloned into pPtGE35 plasmid using Golden Gate assembly (BsaI-HFv2, NEB). The recombinant plasmid was transformed into DH5α to verify the properly assembling. Then, the properly cloned gRNA plasmid was transformed into Epi300 *Escherichia coli* containing pTA-Mob. The plasmid was further transformed into *P. tricornutum* via conjugation from Epi300 *E. coli* mediated by pTA-Mob. The *PtSLC24A* knockouts were screened in Zeocin (100 mg/L) added 1/2 artificial sea water and agarose plate, then verified via PCR amplification and sequencing.

### Measurements of cellular Ca^**2**+^ concentrations

We collected the diatom cells (2 min, 3000 × *g*) and washed twice in Hanks’ Balanced Salt Solution (HBSS, without Ca^2+^, Mg^2+^, and phenol red). Then, the cells were loaded with Fluo-4-AM (1 μM) (HY-101896, MCE) for 1 h at 20°C. After the dye loading, the cells were rinsed with HBSS for three times, then resuspended with ASW (f/2 added) and diluted to different cell densities. Different density cells were treated under WL, Dark, and BL for 24 h. The Ca^2+^ fluorescence (*λ*_ex_ = 494 nm, *λ*_em_ = 516 nm) and cell densities (indicated by OD_730_) were determined using Micro-plate Reader (TECAN Infinite M1000). The Ca^2+^ intensity was normalized with OD_730_, calculated as F516/OD_730_.

To monitor the response of intracellular calcium ions to allelopathic substances, 10 μM Aldehyde (2E, 4E/Z)-decadienal (DD) was added to the culture medium, and the fluorescence of Ca^2+^ (F) was recorded every 20 s. The Ca^2+^ concentration oscillates were indicated with F/F0.

### Apoptosis determination

To investigate the influence of cell density on nuclear morphology, algal cells were subjected to low cell density and high cell density treatments for 24 h. Following centrifugation and washing with PBS, the algal cells were resuspended in sterile seawater. Hoechst 33342 (10 μg/ml) was added for nuclear staining, which binds to double-stranded DNA, with a maximum excitation wavelength of 350 nm and a maximum emission wavelength of 461 nm. Subsequently, fluorescent microscopy was employed for visual observation and size statistics (NIKON NI-U).

Annexin V-EGFP was utilized to detect cells in the early stages of apoptosis post different density treatments. Algal cells were collected by centrifugation, washed, and resuspended in sterile seawater. Annexin V protein pre-labeled with enhanced green fluorescent protein (EGFP, exhibiting strong, and stable fluorescence) was added and thoroughly mixed. The mixture was incubated in darkness at room temperature for 30 min. Flow cytometry (FACS Aria II) was then employed with the FITC module to observe and enumerate cells (excitation wavelength 465–495 nm; emission wavelength 512–558 nm).

### Querying the Tara Oceans eukaryotes gene atlas

To detect the presence of *SLC24A* homologs among the marine eukaryotes, we searched sequences containing Na^+^/Ca^2+^ exchange domain (PF01699) using hmmsearch analysis in both the Marine Atlas of Tara Oceans Unigenes and eukaryotes metatranscriptomes (MATOUv1 + metaT) [60, 61]. The hmmsearch analysis were performed at OGA website (http://tara-oceans.mio.osupytheas.fr/ocean-gene-atlas/), with an e-value <1e−40. The identified homologs were phylogenetic assigned into different taxonomic group according to their taxonomic annotation. The abundance of these *SLC24A* homologs was calculated as the sum of the total gene coverages for each sample [60]. Typically, two depths were sampled in the photic zone: subsurface (SRF) and deep-chlorophyll maximum (DCM). After separating the sites at SRF and DCM by depth, the abundance of samples belonging to the same station is summed. The relative abundance was normalized with Actin (PF00022), which was identified and calculated at the same way described above. The chlorophyll content in each Tara stations comes from the collation of Carradec *et al*. [61], which is also available at PANGAEA. Before correlation analysis, invalid data, including: stations with chlorophyll content of 0, abundance concentration of 0, and a few abnormal outliers, were removed. Correlations between NCX abundance and chlorophyll content among various Tara stations were conducted by GraphPad Prism 8.0.2. The geographic distribution of NCX in marine diatoms was visualized using Origin 2018 software (https://www.originlab.com/).

Thirty-three representative sequences were selected from different taxa to construct the phylogenetic tree using the Neighbor-Joining method in MEGA7 [62]. The percentage of replicate trees was estimated in which the associated taxa clustered together in the bootstrap test (500 replicates).

### Statistical analysis

All statistical data analysis was conducted by PASW statistics 18.0 (http://www.spss.com.hk/statistics/). A one-way ANOVA was carried out to determine the significant difference at *P* < .05.

## Results

### Experimental design and basic information of transcriptome

We designed an orthogonal experiment involving varying cell densities and light conditions to stimulate the release of chemical signals and chlorophyll fluorescence under different cell densities (Fig. 1A). On the one hand, diatoms elicit chlorophyll fluorescence under WL and BL conditions, with fluorescence intensity being directly proportional to cell density [33]. In contrast, chemical cues also correlated with cell density, a phenomenon unaffected by light presence or absence. Consequently, we devised three distinct light environments (WL, BL, and Dark) and established three corresponding cell densities (low, medium, and high) (Fig. 1A). To avoid potential nutrient deficiencies and cell density fluctuations due to prolonged culture, samples were collected after 24 h, and transcriptome analyses were conducted.

In total, 37 143 480–56 004 136 raw reads were obtained among 27 samples using Illumina Novaseq6000 (Table S3). The Q20 ranged from 98.00% to 98.37% and the percentage of the sequenced reference gene ranged from 94.54% to 96.07% (Table S3). All the above demonstrated high sequencing quality of these 27 samples. A total of 11 genes were selected randomly to conduct the qRT-PCR verification (Fig. 1B). There was a significant positive correlation (Pearson r: 0.9656, *P* < .01) between qRT-PCR and RNA-seq (Fig. 1B), proving the consistent expression pattern and the reliability of the transcriptome.

### WGCNA and identification of hub-genes

**Figure 2:**
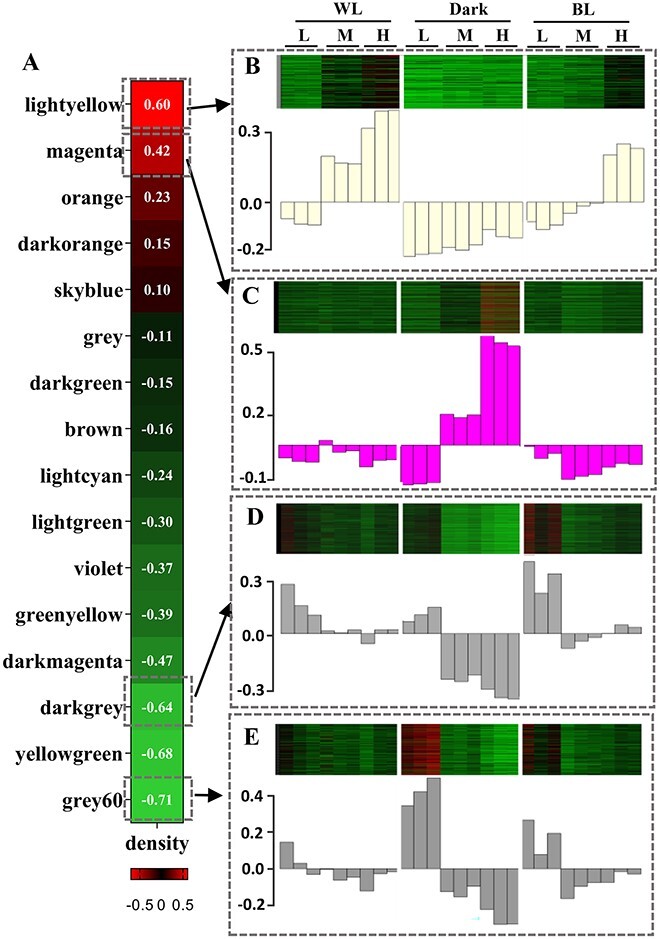
Co-expression modules determined by WGCNA; (A) heatmap of correlations between modules and characteristics (density); expression pattern of the genes and eigengenes of each module: (B) lightyellow; (C) magenta; (D) darkgrey, (E) grey60; the heatmap was plotted using the normalized FPKM values.

To identify the density-dependent co-expressed modules under WL, BL, and Dark conditions, we conducted the WGCNA by screening 10 241 genes in transcriptome. According to the principle of scale-free network, the correlation coefficient and the average connectivity of genes were comprehensively considered to select the appropriate power value (Fig. S1). Based on the correlation between genes and cell density and the similarity of expression patterns within genes, genes were divided into 16 modules. Among them, six modules (lightyellow, magenta, darkmagenta, grey60, yellowgreen and darkgrey) showed significant correlations (|*r*|>0.4) with density (*P* < .05) (Fig. 2A), suggesting strong association with density responses*.*

To further understand the expression pattern of each co-expressed modules, FPKM values of the genes were plotted in a heatmap, and eigengenes expression pattern of each module (equivalent to the module expression pattern) were plotted below the heatmap (Fig. 2B–E). Genes in lightyellow module were induced by high cell density under WL and BL conditions (Fig. 2B), whereas genes in magenta modules only showed density-dependent expression patterns under dark conditions (Fig. 2C). Genes in darkgrey (Fig. 2D) and grey60 (Fig. 2E) modules showed the expression of genes decreased along with increased cell density. In comparison, genes in darkmagenta and lightgreen modules did not show density-dependent expression under WL and BL conditions (Fig. S2). Thus, considering the trait correlation and gene expression in the modules, we selected four modules most representative that are most relevant to cell density: lightyellow, magenta, grey60, and darkgrey, for subsequent analysis.

**Figure 3:**
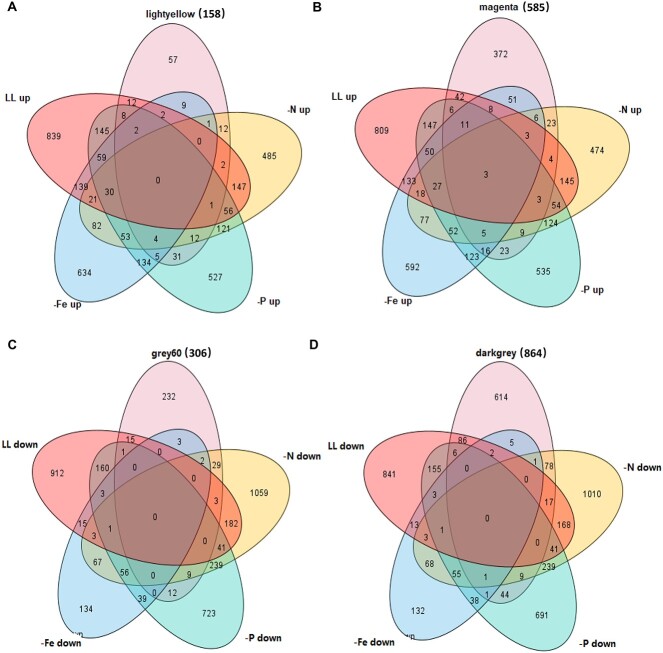
Transcriptome comparison and analysis between density gradient, N limitation (-N), P limitation (-P), Fe limitation (-Fe) and LL; to make a unified comparison, different IDs in the four transcriptomes were mapped and converted into Gene ID, and a few genes that were not mapped were removed; for -P and LL transcriptome, only differentially regulated genes with log2FoldChange > 0.58 or log2FoldChange < −0.58 were included; for -N and -Fe transcriptome, only differentially regulated genes with FoldChange >1.5 or FoldChange <0.67 were included; Venn diagram shows the comparison of DEGs in the above transcriptomes with four modules, including lightyellow (A), magenta (B), grey60 (C), darkgrey (D); the numbers in parentheses are the total number of genes in the module.

Although we designed 24-h treatment to avoid nutrient deficiencies, there is potential concerning that the expression of genes under high cell density may also be influenced by nutritional status and light. To analyze whether our target genes are also affected by these factors, we compared the genes in the lightyellow and magenta modules with the genes that were upregulated in the transcriptome under phosphorus limitation (-P) [63], nitrogen limitation (-N) [64], Fe limitation (-Fe) [65] and low light (LL) [66] conditions. Also, the genes in the darkgrey and grey60 modules were compared with the genes that were downregulated under phosphorus limitation (-P) [63], nitrogen limitation (-N) [64], Fe limitation (-Fe) [65], and LL [66]. After further filtering, there were 57 genes remaining in the lightyellow module (Fig. 3A), 372 genes in the magenta module (Fig. 3B), 232 genes in the grey60 module (Fig. 3C), and 614 genes in the darkgrey module (Fig. 3D). The expression of these genes is solely influenced by cell density.

To have a more intuitive understanding of the density-related biological pathways, the unique genes expressed in the four modules were analyzed by KEGG enrichment analysis. Genes in the lightyellow module were most enriched in mismatch repair pathway (Table 1). In the magenta module, genes were significantly enriched in pathways related to ribosome biogenesis and ribosome (Table 1). Regarding the darkgrey module, glycosylphosphatidylinositol (GPI)-anchor biosynthesis pathway was most enriched (Table 1). Genes in the grey60 module were enriched in lipoic acid metabolism, spliceosome, and nucleocytoplasmic transport pathways (Table 1). These results indicate that intracellular genes are regulated and expressed in different patterns in response to changes in cell density.

**Table 1:** KEGG pathway enrichment analysis of genes that unique expressed in four modules. TOP three KEGG enrichment pathways were shown.

Module	TOP 3 Pathway	Pathway ID	*P* value
lightyellow	Mismatch repair	ko03430	.066132
	DNA replication	ko03030	.102789
	Carbon fixation in photosynthetic organisms	ko00710	.112584
magenta	Ribosome biogenesis in eukaryotes	ko03008	.000001
	Ribosome	ko03010	.002366
	Protein processing in endoplasmic reticulum	ko04141	.012395
grey60	Spliceosome	ko03040	.004967
	Nucleocytoplasmic transport	ko03013	.016418
	Lipoic acid metabolism	ko00785	.055989
darkgrey	GPI-anchor biosynthesis	ko00563	.008852
	Endocytosis	ko04144	.024794
	Spliceosome	ko03040	.033265

**Figure 4:**
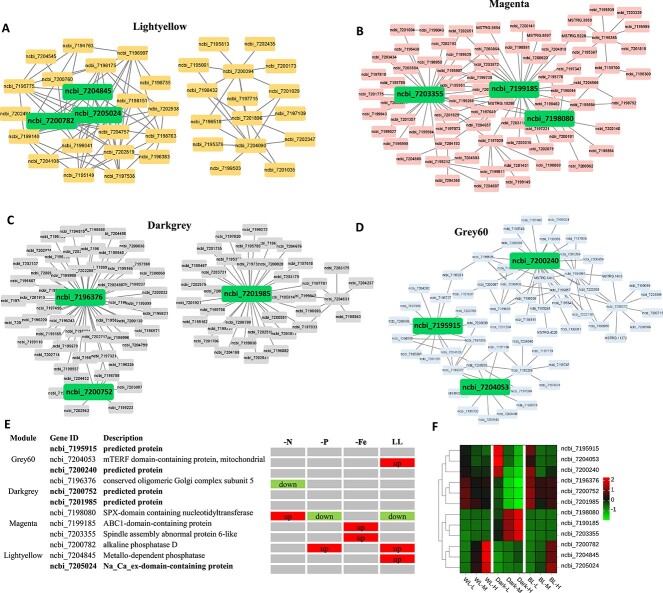
Identification of density-related hub-gene with potential regulatory role; network of genes in four modules: lightyellow (A), magenta (B), darkgrey (C), grey60 (D); the three genes at the pivotal position in the regulatory relationship network are highlighted as the hub genes; the expression pattern of identified hub genes under different environment and cell densities is plotted in (E) and (F).

To identify the hub genes in the four modules, network of the detected co-expressed modules was constructed. Twelve genes located at the network central were identified as the potential hub-gene (Fig. 4A–D). By further analyzing the expression patterns of the 12 hub genes, we found that the expression of the majority hub genes is affected by nutritional status (-N, -P, -Fe) and LL (Fig. 4E). However, *PtSLC24A* (ncbi_7205024), which encode a NCX domain containing protein, remains unaffected by other environmental condition (Fig. 4E) and exhibits a pronounced density-dependent expression (Fig. 4F). Consequently, *PtSLC24A* is the most likely gene which play a pivotal role in perceiving and transmitting cellular density signals.

### Characterization of PtSLC24A and its knockout effects on intracellular Ca^**2**+^ concentration

**Figure 5:**
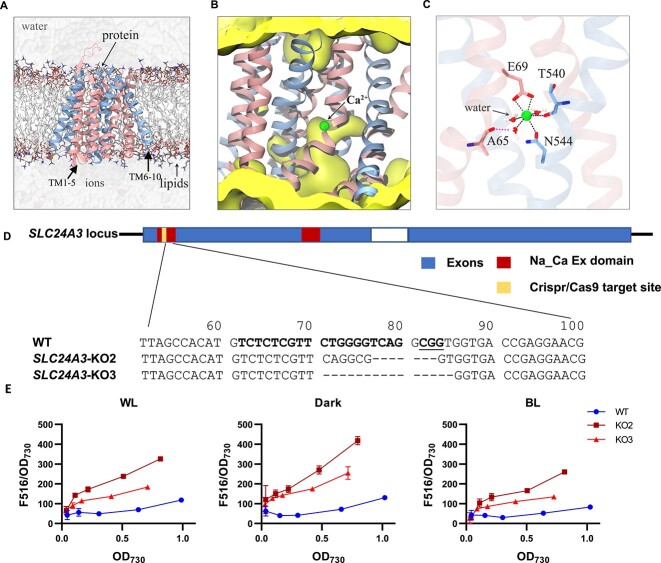
The role of *PtSLC24A* in regulation of Ca^2+^ level and density-dependent genes expression; Ca^2+^-loaded PtSLC24A as suggested by molecular dynamics simulations; (A) all-atom model of PtSLC24A embedded in a lipid membrane with explicit solvent; the N- and C-terminal halves of the transporter across the membrane are depicted in cartoons; the lipids are represented in a stick model, and Na^+^ and Cl^−^ ions as spheres; (B) water-density isosurfaces around the ion-binding region; (C) close-up view of the Ca^2+^ ion binding at the S_Ca_ site in the protein core; S_Ca_ is a Ca^2+^-specific site; the Ca^2+^-coordinating residues are highlighted as sticks; the dashed lines around the Ca^2+^ indicate the coordination bonds, with the water molecules in ball-stick representation; (D) CRISPR/Cas9-induced knock-out mutations at the *PtSLC24A* locus; structure of the *PtSLC24A* locus is shown as the upper column; dashes (-) indicate deletions; DNA sequence chromatogram is shown below the corresponding sequence; (E) intracellular Ca^2+^ concentration stimulated by increased cell density in WT, *PtSLC24A*-KO2, and *Pt SLC24A-*KO3 strains under WL, Dark, and BL conditions.

We modeled the 3D structure of the PtSLC24A protein using the state-of-the-art protein structure prediction method, AlphaFold [47], to explore its function at the molecular level. AlphaFold returned an inward-facing conformation of PtSLC24A in its TM domain that is favorable for Ca^2+^ binding from the cytoplasmic side [46, 48]. With reference to the Ca^2+^-loaded crystal structure of a homogenous NCX from *M. jannaschii* [46, 48], we then placed a Ca^2+^ inside the PtSLC24A TM pore and performed all-atom molecular dynamics simulations on the system with explicit membrane and solvent environment (Fig. 5A). The simulations revealed that the inward-facing PtSLC24A TM domain exhibits a semi-open conformational state with a continuous aqueous channel leading from the intracellular solution to the Ca^2+^ occupancy site (Fig. 5B). Similar to those observed in other membrane transporters [48, 53], the Ca^2+^ is hepta-coordinated in a distorted pentagonal bipyramidal geometry with three amino acid residues (viz. E69, T540, and N544) from PtSLC24A plus three water molecules (Fig. 5C). One of the three molecules is stabilized by A65 via a hydrogen bond. These residues are spatially and functionally akin to T50, E54, T209, and E213 that form the Ca^2+^-binding site in the above *M. jannaschii* NCX. The computational data hence support the Ca^2+^ transport function of PtSLC24A.

We used CRISPR/Cas9 genome editing to generate *PtSLC24A* knockout mutants in *P. tricornutum*. Two *PtSLC24A* knockout mutants were identified with different deletions as a result of different mutagenic nonhomologous end-joining repair events (Fig. 5D).

To investigate whether the cell density change could alter the intracellular Ca^2+^ concentration, *P. tricornutum* cells were loaded with Fluo-4AM dye and adjusted to different cell density under WL, Dark, BL conditions for 24 h. The change in intracellular Ca^2+^ concentration was represented by the relative fluorescence intensity per unit *P. tricornutum* cells (F516/OD_730_). Obviously, the fluorescence intensity of intracellular Ca^2+^ intensity increased with the elevated cell density, both under WL, BL, and dark conditions (Fig. 5E), suggesting the cell density could induce Ca^2+^ responses. However, at the same cell density, Ca^2+^ intensity in *PtSLC24A* knockouts was higher than WT, indicating that the deletion of *PtSLC24A* increased the intracellular Ca^2+^ concentration (Fig. 5E).

DD is a kind of aldehydes which can trigger the intracellular Ca^2+^ transient, resulting in the NO generation and subsequent physiological process [30]. We evaluated whether high cell density and *PtSLC24A* knockout have effect on intracellular Ca^2+^ responses to DD. The results showed that in WL conditions, the magnitude of elevation in the Ca^2+^ pulse in high density treated-cells was higher than that in low density treated-cells, and this trend was consistent in both WT and *PtSLC24A* mutants (Fig. S3). At the low cell density, the magnitude of elevation in the Ca^2+^ pulse in *PtSLC24A* mutants was higher than that in WT, whereas this difference is not present at high densities (Fig. S3). Nevertheless, these results indicate that both *PtSLC24A* knockout and high-density treatments enhance the intracellular Ca^2+^ sensitivity to DD.

### Effect of PtSLC24A knockout on cellular apoptosis

The mechanisms underlying algal bloom demise are diverse including necrosis, and PCD is supposed to be one of the reason why algal bloom demise in the late stage [7], which always accompanied by high cell density. Apoptosis is the main process of PCD and can be characterized by Hoechst staining, metacaspase expression, and Annexins.

**Figure 6:**
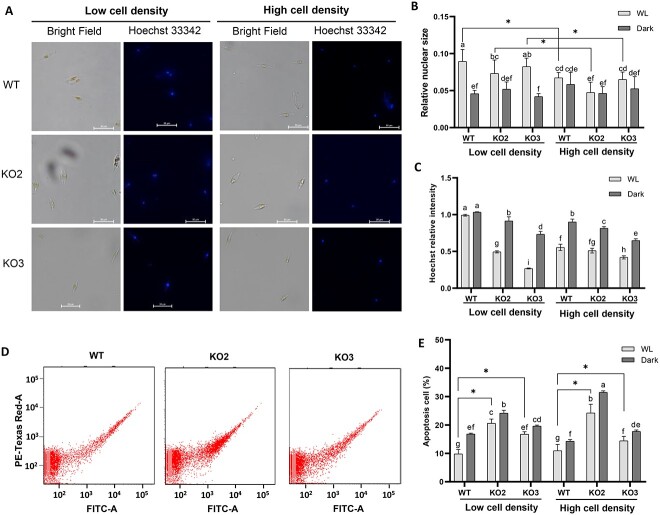
Impact of *PtSLC24A* knockout on cellular apoptosis; *P. tricornutum* nuclear morphology (A), statistical analysis of relative nucleus size (B), and Hoechst intensity (C) following Hoechst 33342 staining after 24 h of low-density and high-density treatments; cell distribution observed using annexin V-EGFP staining and flow cytometry FITC channel (D), along with statistical analysis of apoptosis proportions (E).

Hoechst staining enables the assessment of cell apoptosis by observing changes in nuclear morphology. Normal cell nucleus exhibits a round shape with uniform DNA distribution, resulting in a consistent blue stain. In contrast, apoptotic cell nucleus, due to increased dye concentration, appears bright blue and exhibit a condensed or clumped morphology. Consequently, we quantified the relative size of the cell nucleus compared to the cell to characterize the extent of apoptosis. As depicted in Fig. 6A, cell nuclei become smaller after high-density treatment, indicating an exacerbation of apoptosis. Under high cell density, the *PtSLC24A* knockout nuclei were significantly smaller than those of the WT (Fig. 6B). Also, Hoechst 33342 intensity significantly decreased under high cell density treatment and knockout mutants (Fig. 6C).

Protist lack canonical PCD proteins, including caspases, a family of cysteine-dependent aspartate-directed proteases. However, diatoms encode distant homologues metacaspases, sharing the similar active to execute PCD. Five metacaspase genes have been identified in *P. tricornutum* [67]. Most of the Pt_MC genes (Pt_MC1, Pt_MC2 and Pt_MC3) were upregulated under high cell density (Fig. S6), indicating a density-related PCD in *P. tricornutum*.

Annexins constitute a class of phospholipid-binding proteins widely distributed in the cytoplasm of eukaryotic cells, dependent on Ca^2+^, and participate in intracellular signaling. During the early stages of apoptosis, phosphatidylserine flips from the inner to the outer leaflet of the cell membrane, a critical feature of apoptosis. Annexin V labeled with a green fluorescent probe, EGFP, allows the direct detection of this externalized phosphatidylserine. Overall, high cell density induced the proportion of apoptotic cells, though not significant in WT and *PtSLC24A* KO3 strains. At the same cell density, the proportion of apoptotic cells in the *PtSLC24A* knockout strain is significantly higher than that of the WT (Fig. 6E). These results indicate that high density can induce cell apoptosis, and the knockout of *PtSLC24A* exacerbates this phenomenon.

### Effect of PtSLC24A knockout on the expression of density-related genes

To assess the involvement of *PtSLC24A* in the regulation of density-dependent gene expression, we selected six genes presenting density-dependent expression pattern from transcriptome (Fig. S4) and monitored their transcripts in WT, *PtSLC24A*-KO2, and *PtSLC24A*-KO3 mutants under different cell densities. Overall, the density-dependent expression pattern was maintained in WT, but was diminished or completely abolished in the two *PtSLC24A* loss-of-function lines (Fig. S4), indicating that *PtSLC24A* have an effect on these genes’ expression under different cell densities.

### *In situ*-evidence from Tara oceans

**Figure 7:**
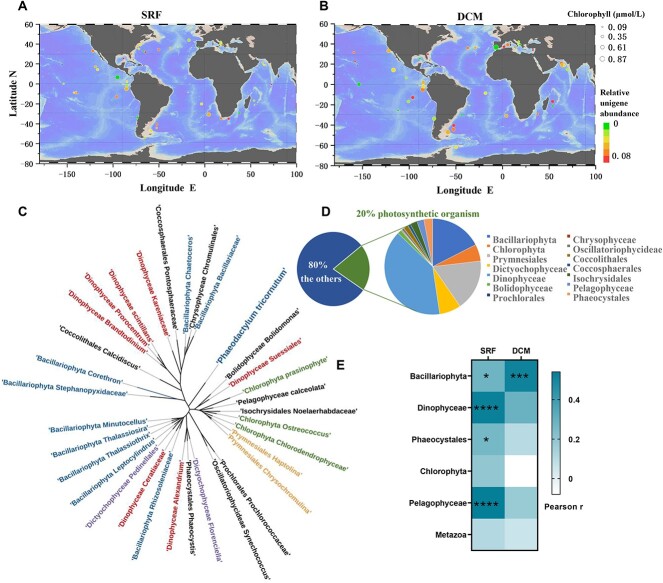
Distribution, phylogenetic analysis, and chlorophyll responsiveness of *SLC24A* in Tara oceans; (A, B) geographic distribution of *SLC24A* in marine diatoms at the surface (SRF, A) and the deep chlorophyll maximum (DCM, B) in Tara Oceans; color scale depicts relative abundance and circle size depicts the chlorophyll content; (C) *SLC24A* evolutionary relationship in marine phytoplankton taxa; the colors represent different taxonomic groups; (D) wide taxonomic distribution of *SLC24A* characterized in Tara Oceans; (E) abundance of *SLC24A* with respect to chlorophyll in different taxonomic groups; color gradient indicates Pearson’s correlation coefficient (Pearson r); significance level is indicated by asterisk (^*^P < .05, ^*^^*^P < .01, ^*^^*^^*^P < .001, ^*^^*^^*^^*^P < .0001).

To assess whether *SLC24A* occurs broadly in marine phytoplankton and what ecological functions phytoplankton *SLC24A* may play, we investigated the occurrence of *SLC24A* homologs which contains NCX domain, in Marine Atlas of Tara Oceans Unigenes and eukaryotes metatranscriptomes (MATOUv1 + metaT). *SLC24A* homologs are present at all sampling stations, no matter SRF or DCM layer, suggesting its wide distribution worldwide (Fig. 7A and B). Also, *SLC24A* homologs are found in all major phytoplankton lineages, of which 20% are photosynthetic organisms and 80% are non-photosynthetic organisms (Fig. 7D). The major marine eukaryotic groups, *Dinophyceae*, *Bacillariophyta*, and *Prymnesiales* accounted for 39.6%, 17.5%, and 17.2% of the photosynthetic organism, respectively (Fig. 7D).

We constructed a phylogeny of 33 representative *SLC24A* homologs derived from Tara Oceans (Fig. 7C). However, the results showed that *PtSLC24A* presented as a single clade and was not closely related to any other taxa, which may be due to the lack of available closely related *SLC24A* homolog sequences in the present database. At the SRF layer, the correlation analysis for *SLC24A* homologs expression with chlorophyll content in Tara Oceans metatranscriptomic data showed that the transcript abundance of the four photosynthetic lineages (*Bacillariophyta*, *Dinophyceae*, *Phaeocystales*, *Pelagophyceae*) was positively correlated with chlorophyll content (Fig. 7E, Fig. S5). At the DCM layer, only *SLC24A* homologs expression in *Bacillariophyta* showed significant positive correlation with chlorophyll content (Fig. 7E, Fig. S5).

## Discussion

### Involvement of Ca^**2**+^ signal in the density-related intracellular signaling mediated by PtSLC24A

At present, it is completely unclear which pathways and which genes are involved in the transmission of density signals within the cell. To address this situation, transcriptome-based WGCNA analysis can rapidly locate candidate hub genes with regulatory roles. Of the candidate hub genes in the center of the co-expression network, only *PtSLC24A* showed a significant dependence on cell density, whereas it remained independent of nutrient status and LL (Fig. 4). Also, along with increasing cell density, the intracellular Ca^2+^ concentration elevated significantly, both under WL, BL, and dark conditions (Fig. 5E). Accordingly, we proposed that *PtSLC24A* plays a regulatory role in the expression level of density-dependent genes by regulating Ca^2+^ concentration. To validate the above hypothesis, we constructed the *PtSLC24A* knockouts and assess their intracellular Ca^2+^ level and density-dependent genes’ transcription. The deletion of *PtSLC24A* increased the intracellular Ca^2+^ level under both WL, Dark, and BL conditions (Fig. 5E). Also, in the two *PtSLC24A* loss-of-function strains, the apoptosis rate increased under high cell density and some of the density-dependent expression pattern was diminished.

The PtSLC24A protein shares varying sequence similarity with the previously reported various eukaryotic and procaryotic NCXs and is annotated as a member of the NCX family. Our computational simulations corroborate the existence of a specific Ca^2+^ binding site within the TM pore of PtSLC24A that is structurally and functionally analogous to that in the NCX from *M. jannaschii* [46, 48]*.* Moreover, the *SLC24A* homologs derived from the main taxonomic groups are ubiquitous globally (Fig. 7A and B), suggesting that the NCX domain is conserved and play a very fundamental and important role throughout the whole marine plankton community. It is well known that tightly controlled changes in cytosolic Ca^2+^ concentration are important for cell signaling in most organisms. Free Ca^2+^ concentration is increased through the activation of a wide range of surface or intracellular Ca^2+^ channels and is returned to resting values through the action of ATP-driven Ca^2+^ pumps located in the plasma membrane and endoplasmic reticulum as well as NCX located on the plasma membrane. As a crucial participant in Ca^2+^ efflux, *PtSLC24A* knockout impedes the process of Ca^2+^ diffusion out of the cell, resulting in the elevation of intracellular Ca^2+^ concentration (Fig. 5E).

The occurrence of some density-dependent physiological responses requires a threshold cell density to start, such as sexual reproduction and resting spore formation [15, 17]. It is obvious that the higher the cell density, the smaller the distance between cells, and the easier it is to achieve intercell communication. Amaral *et al*. introduced the term handover distance to describe the furthest distance cells can communicate with each other [23]. Population of *Pseudo-nitzschia fraudulenta* under darkness showed Ca^2+^-mediated quasi-periodic electrical oscillations, with a handover distance of 120 μm [23]. In this study, the three cell density gradients were 1.5 × 10^6^ cells/ml, 3 × 10^6^ cells/ml, and 6 × 10^6^ cells/ml, corresponding to intercellular distances of 87.36 μm, 69.34 μm, and 55.03 μm, respectively, indicating that the handover distance of density signals is greater than 87.36 μm. However, during diatom blooms in the actual ocean, the cell density could reach 3 × 10^5^ cells/ml [12], with an intercellular distance of 149.38 μm in theoretic. Unlike laboratory conditions, diatom populations in the natural oceans may not exhibit uniform distribution. Turbulent waves and ocean currents could provide more opportunities for cell encounters, facilitating intercellular communication and exchange in a dynamic manner.

Based on the above results, we proposed the PtSLC24A-mediated intracellular signaling mechanism of cell density signals: upon detection of population density signals, such as chemical cues, the TM protein *PtSLC24A* facilitates the efflux of Ca^2+^ ions to tightly control the intracellular Ca^2+^ concentration. This process enables the transmission of intracellular density signals, subsequently governing physiological responses such as cell apoptosis.

The above results worked with model diatom in laboratory, which could facilitate the physiological characterization and molecular mechanism investigation of environmentally important processes, but only partially representative. To link cellular processes at the micro-lab scale to their possible impact on large-oceanic scale, we further explored if this Ca^2+^-dependent density perception mechanism can go into the real global oceans. Metatranscriptomes from 144 Tara Ocean stations were retrieved to analyze the transcriptional abundance of *SLC24A* homologs. A notable and statistically significant positive correlation has been established between the expression of *SLC24A* homologs and phytoplankton biomass in various taxonomic groups, including *Bacillariophyta*, *Dinophyceae*, *Phaeocystales*, *Chlorophyta*, and *Pelagophyceae* within the surface layer (Fig. 7E, Fig. S5). This correlation is consistent with the density-dependent expression pattern demonstrated in controlled laboratory settings. These findings strongly suggest that SLC24A-mediated intracellular Ca^2+^ signaling assumes a crucial role in the signaling mechanisms associated with intracellular population density in natural oceanic environments.

Knocking out *SLC24A* did not completely eliminate the density-dependent expression tendency (Fig. S4), which may be attributed to the multiple and complex intracellular signaling pathways. There may be different signals involved in cell–cell communication and systemic signaling, including reactive oxygen species, lipid derivates, nitric oxide, etc. [6]. Moreover, it is known that the intracellular Ca^2+^ spatiotemporal concentration is affected by various Ca^2+^-related transporters and isoforms, which widely distributed in plasma membrane, mitochondria, and endoplasmic reticulum. Thus, in potential synergistic signaling networks, *PtSLC24A* knockout may not be sufficient enough to completely block intracellular Ca^2+^ signaling for cell density sensing. A very recent and novel hypothesis proposed that environment trigger could elevate intracellular Ca^2+^ concentration, then most Ca^2+^ are expelled out of cells via Ca^2+^ pumps. The elevated extracellular Ca^2+^ concentration formed Ca^2+^ waves and reaches the neighboring cells, acting as an intercellular communication signal [23]. This may also lead to the phenomenon that the density signal cannot be completely blocked though *PtSLC24A* has been knocked out. Therefore, the research on intraspecific communication under the influence of cell density is still in its initial stage. Further in-depth and systematic research on the response mechanism of this process are still needed.

### Density-dependent Ca^**2**+^ signals are regulated by chemical cues, not chlorophyll fluorescence signals

Cell density changes could be sensed by diatoms in the form of chemical cues and chlorophyll fluorescence signals [15–17, 33, 34]. In our previous study, WL and BL could excite the chlorophyll fluorescence in marine diatoms, whereas the dark conditions could not. And the excitation intensity of endogenous chlorophyll fluorescence was enhanced by increased cell density [33]. So, the expression of genes regulated by chlorophyll fluorescence was correlated with cell density under WL and BL, whereas it showed no change under dark conditions [33]. Conversely, the density-dependent expression of genes under the influence of chemical signals was unaffected by light presence or absence. This characteristic serves as a valuable tool for preliminary distinguishing genes and physiological pathways subject to distinct forms of density-mediated signaling.

Based on this expression paradigm, genes were divided into different modules. In contrast to the expression patterns of genes regulated by chlorophyll fluorescence, if the expression level of a gene correlated with cell density both under WL, BL, and dark, it is most likely to be regulated by chemical signals.

In this study, the intracellular Ca^2+^ concentration increased as a function of cell density in *P. tricornutum* (Fig. 5E), both under light or dark. Similarly, *PtSLC24A* also exhibits a density-dependent expression pattern. This expression pattern is consistent with the expression pattern of genes mediated by chemical cues, suggesting that intracellular Ca^2+^ signals are mediated by chemical cues rather than chlorophyll fluorescence signals when density changes.

Previous studies indicate that diatoms could detect and respond to physicochemical changes in their environment, such as phosphorus sensing, iron sensing, and osmotic stress by using sophisticated perception systems based on Ca^2+^ fluctuation [30, 68–70]. Recent studies extended the regulatory role of Ca^2+^ to the cell–cell communication mediated by chemical cues. For example, exogenous N-acyl homoserine lactone, a signal molecules bacterial, could trigger cellular Ca^2+^ efflux in diatoms [31]. In the studies and the latest reviews conducted by Rocha *et al*., upon direct exposure to environmental stimuli such as infochemicals, microalgal cells can generate extracellular Ca^2+^ waves, serving as a signal to transmit and amplify environmental information within the population [23, 32]. Thus, for cells, the received signals may encompass both chemical signals and electrical signals based on Ca^2+^ waves, with the latter also originating from chemical signals. Although our investigation did not ascertain the precise chemical signal induced by cell density, as previously noted, the density-dependent Ca^2+^ signal is independent of light, which strongly implies a chemical cues mediation (Fig. 5E). Consequently, drawing from the evidence derived from previous study and this study, we propose that the intracellular Ca^2+^ signaling pathway may respond to diverse chemical signals in a conserved manner.

Besides chemical cues, we characterized a cluster of genes showing density-dependent pattern mediated by chlorophyll fluorescence signals. In light-yellow module, genes show no density-dependent expression patterns under dark conditions, but under WL and BL, their expression upregulates as the density increases (Fig. 2B). The opposite expression trend appears in the magenta module (Fig. 2C). These expression patterns largely ruled out the mediation of chemical factors, indicating that the density-induced responses of these genes are under the control of chlorophyll fluorescence. Overall, transcriptome results suggested that, besides inducing iron assimilation and coordinate population behavior [33, 34], the chlorophyll fluorescence-mediated signaling pathway has a broad impact on multiple cellular biological processes. However, the grouping situation obtained according to the algorithm is not very accurate, for example, *PtSLC24A* gene, although it is classified in the lightyellow module, but it shows a typical expression pattern of chemical signals.

### Ecological implications of SLC24A role in determining the fate of algal blooms by manipulating PCD

Phytoplankton are subjected to numerous stresses during bloom succession. These stresses include infection by pathogens (viruses, bacteria and parasites), grazers (macro and microzooplankton), and lack of resources (e.g. light, nutrients), which eventually lead to the bloom demise [71]. PCD was one of the most important mechanisms of bloom demise, and suggested to be related to the high turnover rates of phytoplankton [7, 25].

The induction stress of autocatalytic PCD include infochemicals, cell age, nutrient deprivation, high light, excessive salt, oxidative stress, virus invasion, and other stresses [25]. In this study, we observed that elevated cell density effectively triggered the proportion of PCD in diatom cells (Fig. 6). The responses of PCD cascade to DD also accompanied by Ca^2+^ fluxes [30]. Similarly, the high cell density-induced PCD cascade also involves increased Ca^2+^ intensity (Fig. 5E, Fig. 6). Knocking out *PtSLC24A* disrupts calcium efflux, elevating intracellular calcium and intensifying PCD (Figs 5 and 6). Hence, Ca^2+^ is evidently the key component that enables the interplay between intracellular cell density signaling and PCD cascade. Moreover, PtMCA-IIIc is a Ca^2+^-dependent metacaspase, proposed to be involved in PCD regulation in *P. tricornutum* [67]. We observed upregulated *PtMCA-IIIc* under high cell density (Fig. S6), which maybe the promotion of high Ca^2+^ concentration at high cell density. Also, overexpression of *PtMCA-IIIc* led to increased sensitivity to DD [72], while our results showed elevated sensitivity to DD at high cell density (Fig. S3). These results confirmed each other and further indicate that cell density plays a regulatory role in *PtMCA-IIIc* expression by manipulating intracellular Ca^2+^ concentration.

Based on the above analysis, we proposed the mechanism of increased PCD cascade at high cell density: as the dense bloom reaches a high population density, the resultant density signals are sensed by diatom cells, which increase the intracellular Ca^2+^ concentration. Furthermore, the increased intracellular Ca^2+^ stimulated the upregulation of PtMCA-IIIc, finally accelerating the PCD cascade. During which, *SLC24A* affects the PCD cascade by manipulating the intracellular Ca^2+^ level.

By eliminating damaged cells from a population and providing surviving cells with limiting nutrients, PCD in unicellular organisms appears to confer heightened genetic and population fitness, serving as an adaptive mechanism beneficial to the overall population [25]. This process could significantly influence bloom fate by manipulating cellular fate [30]. Our findings offer a new perspective for understanding the factors that condition the phytoplankton bloom dynamics. Nevertheless, in comparison to other extensively researched factors, the contribution of density signals-induced PCD in bloom demise needs to be further evaluated.

## Conclusion

In this study, we designed an orthogonal experiment involving cell density and light field to investigate key components responsible for perceiving and transmitting intracellular cell density signals. Through transcriptome sequencing combined with bioinformatics analysis, we identified the *SLC24A*, encoding a Na^+^/Ca^2+^ exchange domain-containing protein. When the population density fluctuates, the *SLC24A* regulates intracellular Ca^2+^ levels by controlling the efflux of Ca^2+^ as cell sense chemical cues, thereby maintaining PCD cascade at the specific level. The detection of cell density mediated by intracellular Ca^2+^ signaling mechanism provides insights into the cell–cell communication during diatom blooms. However, further studies are required to identify the potential infochemicals and gain a better understanding about the underlying molecular machenism.

## Supplementary Material

240227-supplementary_file-Figure_S1_wrae039

240227-supplementary_file-Figure_S2_wrae039

240227-supplementary_file-Figure_S3_wrae039

240227-supplementary_file-Figure_S4_wrae039

240227-supplementary_file-Figure_S5_wrae039

240227-supplementary_file-Figure_S6_wrae039

Table_S1_wrae039

Table_S2_wrae039

Table_S3_wrae039

## Data Availability

The RNA-seq raw data that support the findings of this study have been deposited into Sequence Read Archive (SRA) of National Center for Biotechnology Information (NCBI) with accession number PRJNA1033529. All secondary derived data and scripts have been deposited to the Zenodo repository with a DOI: 10.5281/zenodo.10730366. The three *P. tricornutum* strains used in this study (WT, *PtSLC24A-*KO2, *Pt SLC24A-*KO3) have been deposited to the Freshwater Algae Culture Collection at the Institute of Hydrobiology (FACHB) with accession number FACHB-3582, FACHB-3583 and FACHB-3584.
