# Supplementary material for: SLC24A-mediated calcium exchange as an indispensable component of the diatom cell density-driven signaling pathway
Source: ISME J. 2024 Mar 8;18(1):wrae039. doi: 10.1093/ismejo/wrae039 (PMC10982851; doi:10.1093/ismejo/wrae039)
Supplement: 240227-supplementary_file-Figure_S1_wrae039 [file 240227-supplementary_file-figure_s1_wrae039.pdf]

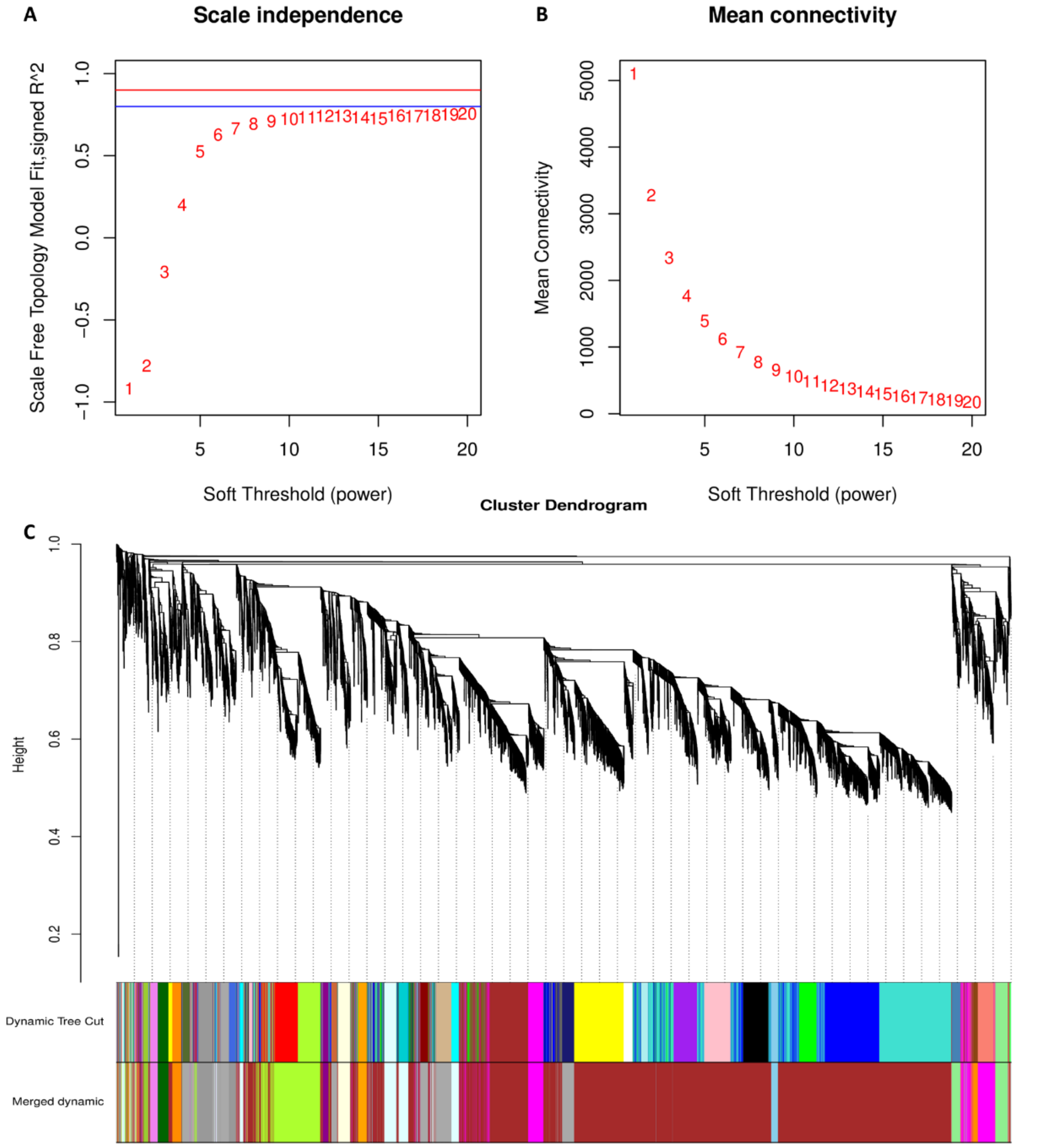


Fig. S1 Co-expression modules determined by weighted gene co-expression network analysis (A). Analysis of the scale-free fit for various soft-thresholding powers. (B). Analysis of the mean connectivity for various soft-thresholding powers. (C). Cluster dendrogram of genes in the WGCNA.
