# Supplementary figures and images for: SLC24A-mediated calcium exchange as an indispensable component of the diatom cell density-driven signaling pathway
Source: ISME J. 2024 Mar 8;18(1):wrae039. doi: 10.1093/ismejo/wrae039 (PMC10982851; doi:10.1093/ismejo/wrae039)

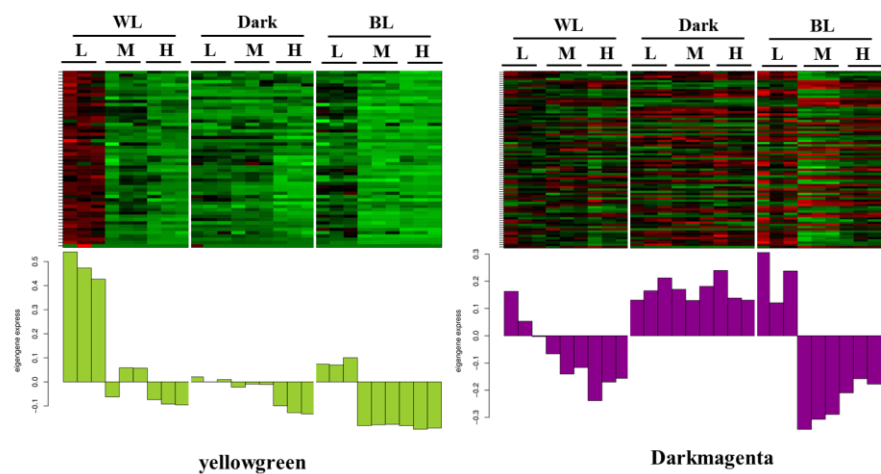

Fig. S2 Expression pattern of the genes and eigengenes of yellowgreen and darkmagenta module.

Supplement: 240227-supplementary_file-Figure_S2_wrae039 [file 240227-supplementary_file-figure_s2_wrae039.pdf]

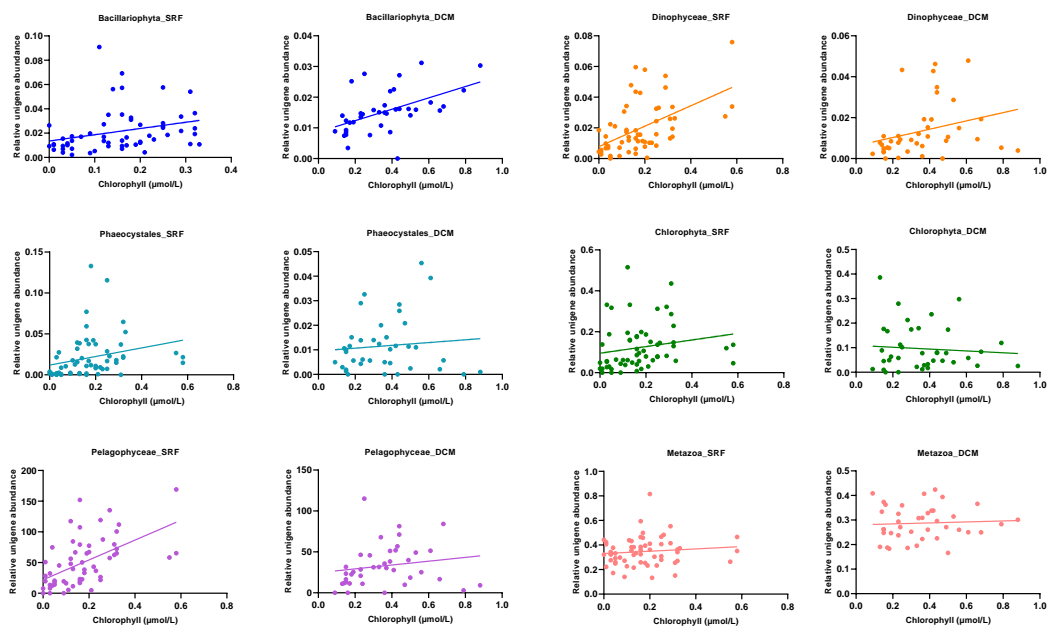

Fig. S5 Abundance of *SLC24A* with respect to chlorophyll in different taxonomic groups.

Supplement: 240227-supplementary_file-Figure_S5_wrae039 [file 240227-supplementary_file-figure_s5_wrae039.pdf]
