# Supplementary material for: SLC24A-mediated calcium exchange as an indispensable component of the diatom cell density-driven signaling pathway
Source: ISME J. 2024 Mar 8;18(1):wrae039. doi: 10.1093/ismejo/wrae039 (PMC10982851; doi:10.1093/ismejo/wrae039)
Supplement: 240227-supplementary_file-Figure_S3_wrae039 [file 240227-supplementary_file-figure_s3_wrae039.pdf]

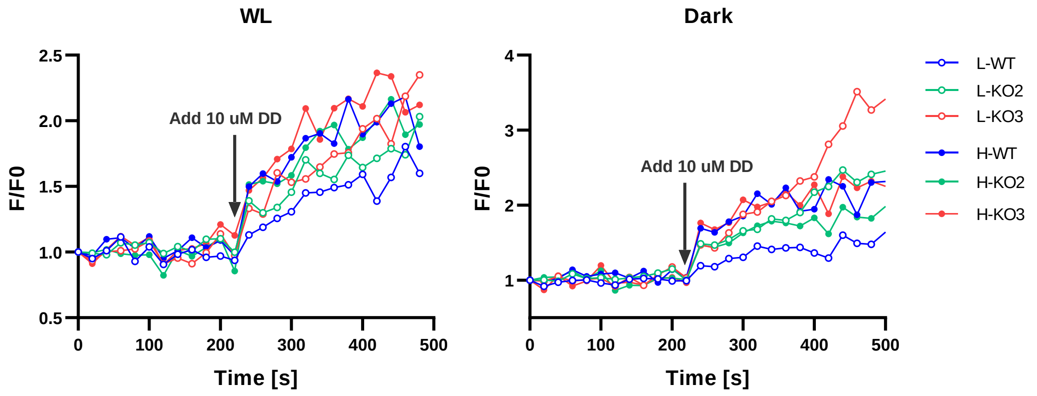

Fig. S3 Effect of aldehyde (2E,4E/Z)-decadienal (DD) on the Ca^{2+} fluctuation in *P. tricornutum*. WT, *PtSLC24A*-KO2 and *PtSLC24A*-KO3 cells were exposed to different cell density (L, M, H) for 24 h. The time-dependent intensity of Ca^{2+} fluorescence was recorded and indicated with F/F0.
