# Supplementary material for: SLC24A-mediated calcium exchange as an indispensable component of the diatom cell density-driven signaling pathway
Source: ISME J. 2024 Mar 8;18(1):wrae039. doi: 10.1093/ismejo/wrae039 (PMC10982851; doi:10.1093/ismejo/wrae039)
Supplement: 240227-supplementary_file-Figure_S4_wrae039 [file 240227-supplementary_file-figure_s4_wrae039.pdf]

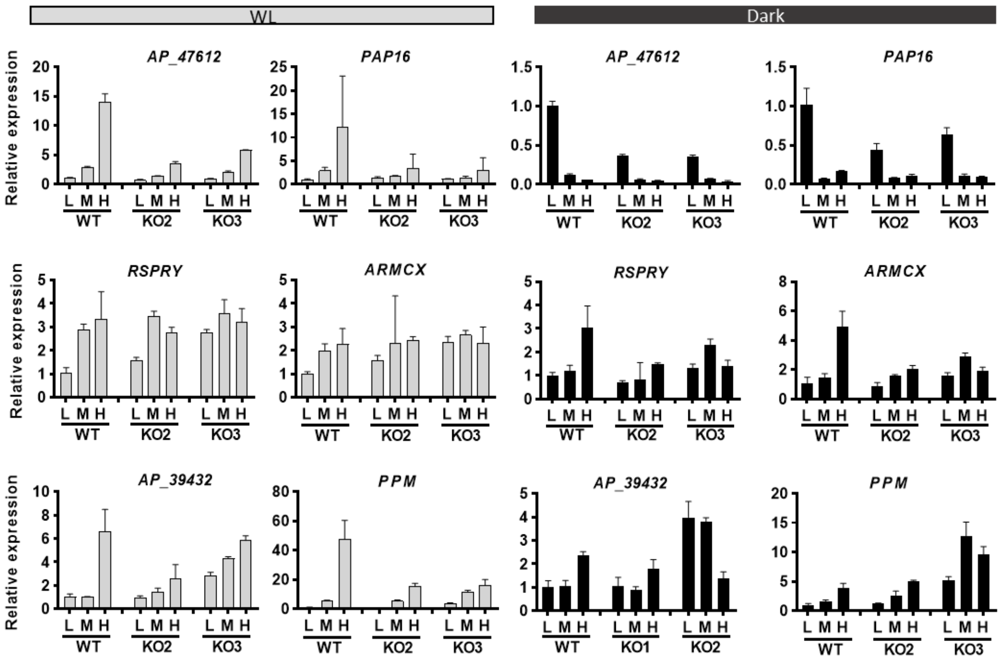

Fig. S4 qRT-PCR analysis of the 6 genes under WL and Dark conditions. WT, *PtSLC24A*-KO2 and *PtSLC24A*-KO3 cells were exposed to different cell density (L, M, H) for 24 h.
