# Supplementary material for: SLC24A-mediated calcium exchange as an indispensable component of the diatom cell density-driven signaling pathway
Source: ISME J. 2024 Mar 8;18(1):wrae039. doi: 10.1093/ismejo/wrae039 (PMC10982851; doi:10.1093/ismejo/wrae039)
Supplement: 240227-supplementary_file-Figure_S6_wrae039 [file 240227-supplementary_file-figure_s6_wrae039.pdf]

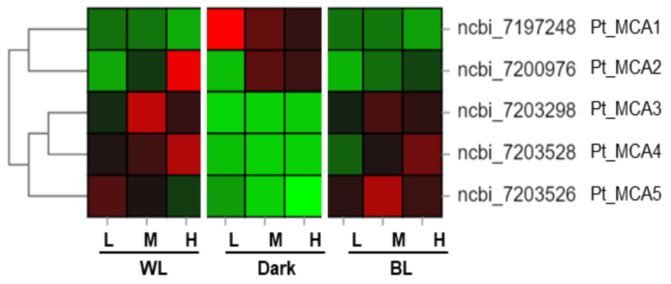

Fig. S6 Transcriptional patterns of 5 metacaspases in the *P. tricornutum* under different cell densities.
